# Conformational determinants of glucagon stability and aggregation kinetics in aqueous and commercial formulations

**DOI:** 10.1002/pro.70750

**Published:** 2026-08-05

**Authors:** Angelo Santoro, Marco Macis, Michela Buonocore, Antonio Ricci, Anna Maria D'Ursi

**Affiliations:** ^1^ Department of Pharmacy University of Salerno Salerno Italy; ^2^ Fresenius Kabi iPSUM Rovigo Italy; ^3^ Department of Chemical Sciences University of Naples “Federico II,” Complesso Universitario Monte Sant'Angelo Naples Italy

**Keywords:** conformational stability, glucagon, peptide aggregation, peptide formulation, structure–stability relationship

## Abstract

Glucagon is a well‐established therapeutic peptide, widely used to treat hypoglycemia. Like many peptide drugs, it offers advantages such as specificity, biocompatibility, and high affinity for receptor targets, but suffers from limited physical and chemical stability. In particular, improper conditions can promote the formation of amyloid fibrils, leading to loss of biological activity and, in some cases, cytotoxicity. Understanding the conditions that modulate the structural behavior of glucagon is therefore crucial. Currently, two injectable formulations are available on the market: a lyophilized vial of glucagon with lactose at acidic pH, and a more recent auto‐injector ready‐to‐use (RTU) formulation containing glucagon in dimethyl sulfoxide (DMSO) with trehalose. In this study, we investigated the conformational properties of glucagon in these two marketed formulations and compared them with glucagon dissolved in aqueous solution at pH 3.5, a metastable condition prone to aggregation. Preliminary circular dichroism and fluorescence spectroscopy were used to confirm glucagon stability over time; subsequently, NMR analysis showed that structural destabilization consistently begins at the C‐terminal region, while the ^11^Ser‐Leu^14^ segment remains structured across all environments. These findings highlight two key determinants of glucagon stability and aggregation. Strategies that preserve the integrity of the C‐terminal region while stabilizing the ^11^Ser‐Leu^14^ motif may improve peptide solubility and extend shelf life, providing a rational basis for the design of next‐generation glucagon formulations for emergency use.

## 1 INTRODUCTION

Glucagon is a 29‐residue peptide hormone (HSQGTFTSDYSKYLDSRRAQDFVQWLMNT), secreted mainly by the α‐cells of the pancreatic islets of Langerhans (Sanchez‐Andres et al., 2026; Zhang et al., 2025). Physiologically, glucagon plays a key role in glucose homeostasis by counteracting the action of insulin (Amin & Salman, 2025; Wu et al., 2025). In fact, it is released in response to hypoglycemia and stimulates hepatic glycogenolysis and gluconeogenesis, thereby increasing blood glucose levels to maintain normoglycemia (Colberg, 2022; Venugopal et al., 2023). Clinically, glucagon is one of the best‐known peptide drugs, primarily used to treat severe hypoglycemia, particularly in insulin‐treated diabetic patients when oral carbohydrate intake is not possible (Algeffari, 2025; Hirsch et al., 2024). Its therapeutic action directly complements insulin therapy, restoring glucose levels when insulin‐induced hypoglycemia occurs (Capozzi et al., 2022; Kajani et al., 2024). However, glucagon exhibits significant solubility issues in water, with pH, temperature, and salt concentration playing critical roles (Beaven et al., 1969; Gratzer et al., 1968). In particular, having an isoelectric point of ~7.0, glucagon exhibits limited solubility at neutral pH. At acidic pH (≤ 3), it initially dissolves but subsequently undergoes gelation driven by fibrillar assembly (Chou & Fasman, 1975; Selkoe, 2003). As a result, glucagon solutions typically exhibit a pH of 2.5–3.0, and maintaining this acidic environment is critical to preserving the formulation's structural integrity and pharmaceutical quality. The solubility of glucagon formulations is often improved by the addition of lactose or derivatized cyclodextrins, which exert a favorable effect on the stabilization of the peptide and its capacity to improve formulation features during both manufacturing and storage (Fang et al., 2012; Mahdizade Ari et al., 2023). The poor solubility of glucagon is a major obstacle to developing soluble formulations that are easy to administer, stable, and have a shelf life of up to 2 years (Pisano et al., 2024). Consequently, glucagon formulations are commonly manufactured and commercialized as lyophilized powders that require reconstitution in acidic media immediately before administration (Kumar et al., 2024). This requirement poses significant challenges during hypoglycemic crises, where rapid intervention is essential and ready‐to‐use formulations would be highly desirable. To address this unmet need, a glucagon formulation dissolved in dimethyl sulfoxide (DMSO), commercially known as GVOKE, has been developed and FDA‐approved (Sherman & Lariccia, 2020). However, due to the intrinsic toxicity of DMSO, this formulation is intended for emergency use only and is not suitable for prolonged use or implantation. This restriction is particularly relevant considering the increasing interest in dual‐hormone delivery devices that can automatically administer both insulin and glucagon in closed‐loop systems (Blauw et al., 2016; Haidar et al., 2015; Russell et al., 2014). To develop a stable and safe glucagon solution, it is crucial to understand the structural preferences of glucagon in physiological solution and the factors that influence its transition from soluble forms to fibrillar aggregates. Structural studies showed that glucagon readily aggregates into amyloid fibrils, a process that can impair its biological activity and induce cytotoxic effects in neuronal cells in vitro (Murphy, 2002; Onoue et al., 2004; Pedersen, 2010; Santoro, Ricci, et al., 2025; Zapadka et al., 2017). Solubilized glucagon can adopt two different states, leading to fibrils with distinct morphologies. At concentrations below 1 mg/mL, glucagon mainly exists as unstructured monomers that tend to form twisted fibrils, and oligomers that form untwisted fibrils (Košmrlj et al., 2015). In contrast, at concentrations above 1 mg/mL, glucagon assembles into trimers and oligomers. Across all concentrations, fibril elongation alternates between periods of growth (“go” state) and stasis (“stop” state) (Košmrlj et al., 2015). Remarkably, fibril growth is most pronounced at both low and high concentrations, but reduced at intermediate levels (around 3 mg/mL) (Andersen et al., 2007). Nuclear magnetic resonance (NMR) studies in solution have shown that in lipid‐water interfaces, glucagon adopts partially ordered α‐helices, mainly within the N‐terminal region, while the C‐terminal residues remain flexible (PDB ID: 1KX6; Braun et al., 1983). This suggests that membrane‐like surfaces can stabilize amphipathic helices and modulate peptide‐lipid interactions, potentially influencing aggregation (Braun et al., 1983). Studies on glucagon antagonist [desHis^1^, desPhe^6^, Glu^9^]glucagon amide, investigated in dodecyl phosphocholine (DPC), support the presence of an amphipathic two‐helix motif arranged in an “L‐shape” orientation (PDB ID: 1NAU; Ying et al., 2003). Despite the structural modifications, the conformational features highlight intrinsic structural propensities of glucagon‐like sequences when exposed to micellar or membrane‐mimetic environments. Such results point to a delicate equilibrium between disordered states and transient α‐helices, whose stabilization or disruption may determine whether the peptide remains soluble or progresses toward aggregation (Ying et al., 2003). Similar behavior has been observed in other amyloidogenic peptides, such as α‐synuclein, in which non‐fibrillar N‐terminal regions can modulate aggregation kinetics (Skaanning et al., 2020). On the other hand, solid‐state NMR analysis of glucagon fibrils reveals a β‐strand‐rich structure with two main conformations, predominantly in the central and C‐terminal regions. This aligns with previous findings that these regions promote self‐assembly through intermolecular hydrogen bonds (PDB ID: 6NZN; Gelenter et al., 2019). To enhance the comprehensiveness of the structural data, this study investigates the behavior of glucagon within a metastable solution environment and in two commercially available formulations. The metastable environment consists of an aqueous solution at pH 3.5, which favors early aggregation. Regarding the formulations, we selected (i) an aqueous solution containing lactose and HCl at pH 2.5, and (ii) a DMSO solution supplemented with H_2_SO₄ and trehalose dihydrate, which corresponds to the composition of the aforementioned GVOKE. Structural characterization was performed through circular dichroism (CD) and fluorescence spectroscopy, followed by the acquisition of solution NMR experiments to define conformational features and aggregation tendencies. By comparing these environments, we aim to identify the structural determinants that promote either soluble conformations or transitions to fibrillar assemblies, thereby providing insights relevant to the rational design of stable glucagon formulations.

## 2 RESULTS

For clarity, in this study we adopted the following nomenclature:

- Solution A: 1 mg/mL glucagon in aqueous solution at pH 3.5;
- Formulation B: 1 mg/mL glucagon in aqueous solution, in the presence of 107 mg/mL lactose, at pH 2.5;
- Formulation C: 5 mg/mL glucagon in DMSO, in the presence of 56 mg/mL trehalose dihydrate and 6 mg/mL sulfuric acid (1N H_2_SO_4_).

### 2.1 Circular dichroism

**FIGURE 1:**
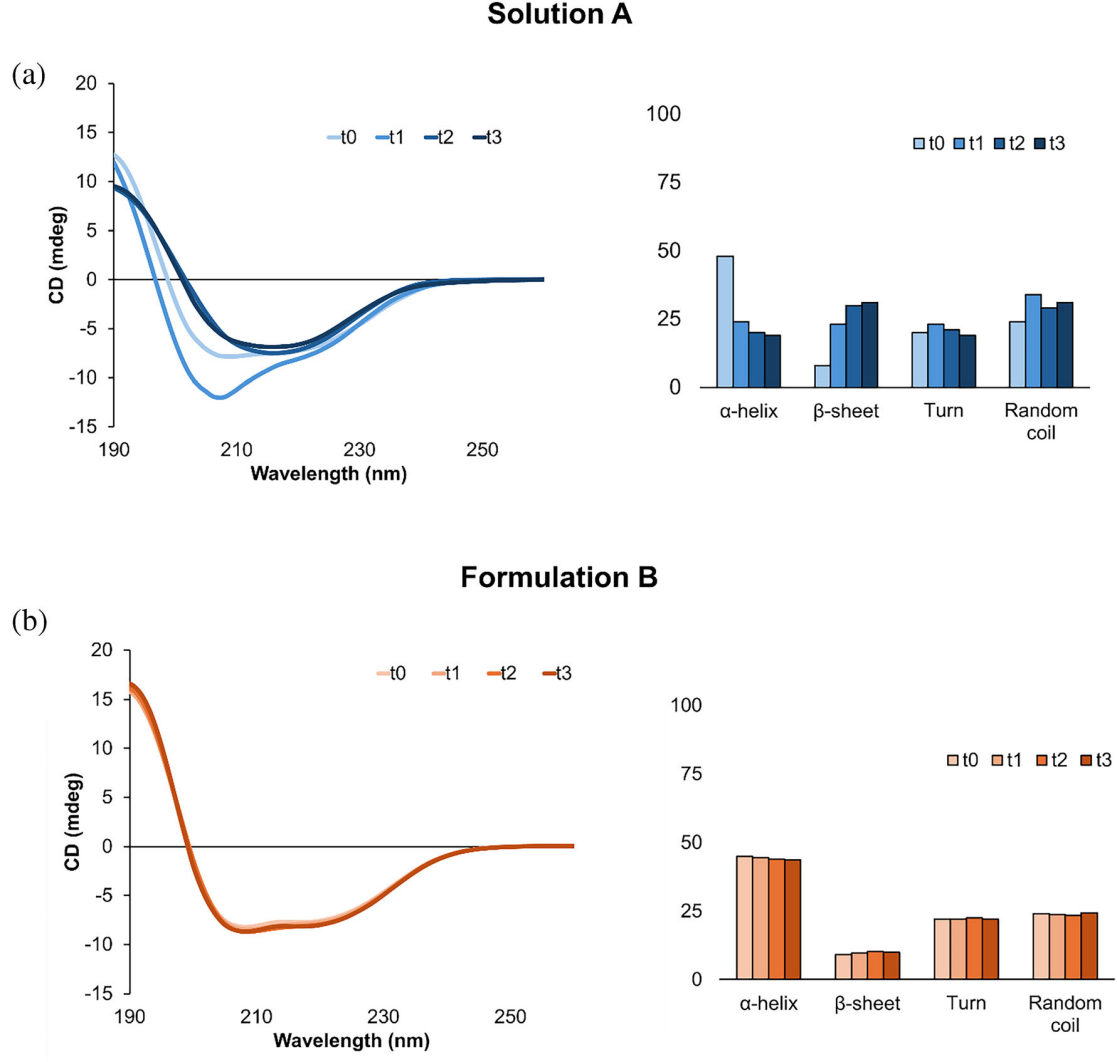
CD curves and secondary structure quantification performed with the CONTIN algorithm of (a) glucagon in water at pH 3.5 (Solution A) and (b) glucagon in water and lactose at pH 2.5 (Formulation B) at system establishment (t0) and after one (t1), two (t2) and three (t3) days.

CD spectra were collected on the aqueous systems of glucagon (Solution A and Formulation B). Formulation C could not be analyzed because of spectral interference from DMSO, whose intrinsic absorption profile overlaps with the far‐UV region (190–240 nm) (Cotton et al., 1999). For Solution A and Formulation B, CD analysis was performed at time zero (t0) and after 1 (t1), 2 (t2), and 3 days (t3) to monitor any conformational change over time. Figure 1 shows the CD spectra for both solutions. In Solution A, the peptide initially assumes a helix conformation (t0). After 1 day (t1), the CD spectrum reveals a partial loss of the helix component with a concomitant increase in β‐sheet and unordered structures. At subsequent time points (t2 and t3), the helix content progressively decreases, while β‐sheet features become more prominent. Quantitative deconvolution of the spectra using the CONTIN algorithm on the DICHROWEB platform (Whitmore & Wallace, 2004) supported these observations (Figure 1a). In Formulation B, glucagon displayed a predominant helix structure at t0, which remained essentially unchanged over the 3‐day monitoring period, consistent with improved conformational stability under these conditions (Figure 1b).

The stability of the helix structure was further assessed by analyzing the intensity ratio of the two α‐helix characteristic minima (220/208 nm). A ratio close to 1 indicates a well‐preserved helical conformation. In Solution A at t0, as well as in Formulation B at all time points, this ratio was approximately 1, suggesting structural stability. However, in Solution A at t1, the ratio dropped below 1, consistent with a more flexible or partially destabilized helix, anticipating subsequent conformational loss (Greenfield, 2004; Santoro, Buonocore, & D'Ursi, 2025).

### 2.2 Fluorescence analysis

**FIGURE 2:**
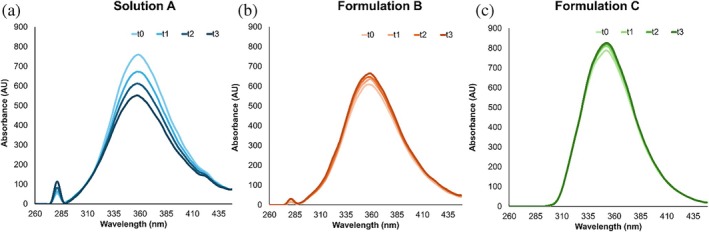
Fluorescence curves of (a) glucagon in water at pH 3.5 (Solution A, blue gradient), (b) glucagon in water and lactose at pH 2.5 (Formulation B, orange gradient) and (c) glucagon in DMSO, in the presence of trehalose and sulfuric acid (Formulation C, green gradient) at system establishment (t0) and after one (t1), two (t2) and three (t3) days.

Fluorescence measurements were performed to support CD analysis (Housmans et al., 2023; Xiong et al., 2021). The intrinsic fluorescence observable in peptides mainly derives from the aromatic residue of tryptophan (Trp), which emits fluorescence following excitation with UV light, due to the presence of an indole ring (Lakowicz, 2006), and to a lesser extent, from tyrosine (Tyr) and phenylalanine (Phe). The emission wavelength of these residues is sensitive to their microenvironment, shifting according to transitions between buried hydrophobic regions and solvent‐exposed states (Vivian & Callis, 2001). Figure 2 reports the fluorescence spectra of glucagon in Solution A (Figure 2a), Formulation B (Figure 2b), and Formulation C (Figure 2c) recorded at t0, t1, t2, and t3. In Solution A, the fluorescence emission maximum at 360 nm was observed at t0, suggesting that the Trp indole group was exposed to the aqueous environment. Over time, the emission intensity decreased, indicative of conformational rearrangements that altered solvent exposure (Figure 2a) (Burstein et al., 1973). In formulations B and C, the fluorescence spectra remained relatively unchanged between t0 and subsequent times, indicating structural stability likely due to the solvent system (Figure 2b,c).

The potential formation of early‐stage fibrillar aggregates was monitored by a classical Thioflavin T (ThT) fluorescence assay. The samples were observed for 3 days, incorporating early intermediate time points at 2 and 6 h to trace the initial phases of structural transition. Remarkably, the ThT emission profiles revealed highly divergent behaviors among the tested systems: Solution A exhibited a progressive and time‐dependent increase in ThT fluorescence intensity, confirming the rapid assembly of the peptide into fibrils in this metastable environment (Figure S1A). On the other hand, Formulations B and C showed different behavior, with low ThT fluorescence throughout the 72‐h monitoring window (Figure S1B,C), confirming the stability of the soluble peptide in these environments. Additionally, we simulated long‐term aging of glucagon to predict its physical stability over shelf‐life scales otherwise inaccessible during standard experimental timeframes. The samples were subjected to accelerated stress conditions by incubating them at 37°C under continuous mechanical shaking at 900 rpm for up to 48 h. The conformational integrity of the peptide was monitored through intrinsic tryptophan fluorescence spectroscopy. Under these conditions, Solution A showed a drastic and immediate drop in emission intensity accompanied by a severe blue shift of the emission maximum within the first 30 min of stress, confirming an instantaneous structural collapse and the burial of aromatic fluorophores (Figure S2A). Formulation B exhibited a noticeable blue shift and a significant alteration in its intrinsic fluorescence profile after 2 h, which then persisted over time (Figure S2B), suggesting that prolonged thermo‐mechanical stress can eventually breach the thermodynamic protective barrier of this aqueous environment. On the contrary, Formulation C demonstrated exceptional integrity, with the absence of any shift or intensity loss throughout the entire 48 h shaking period (Figure S2C).

### 2.3 NMR analysis

**FIGURE 3:**
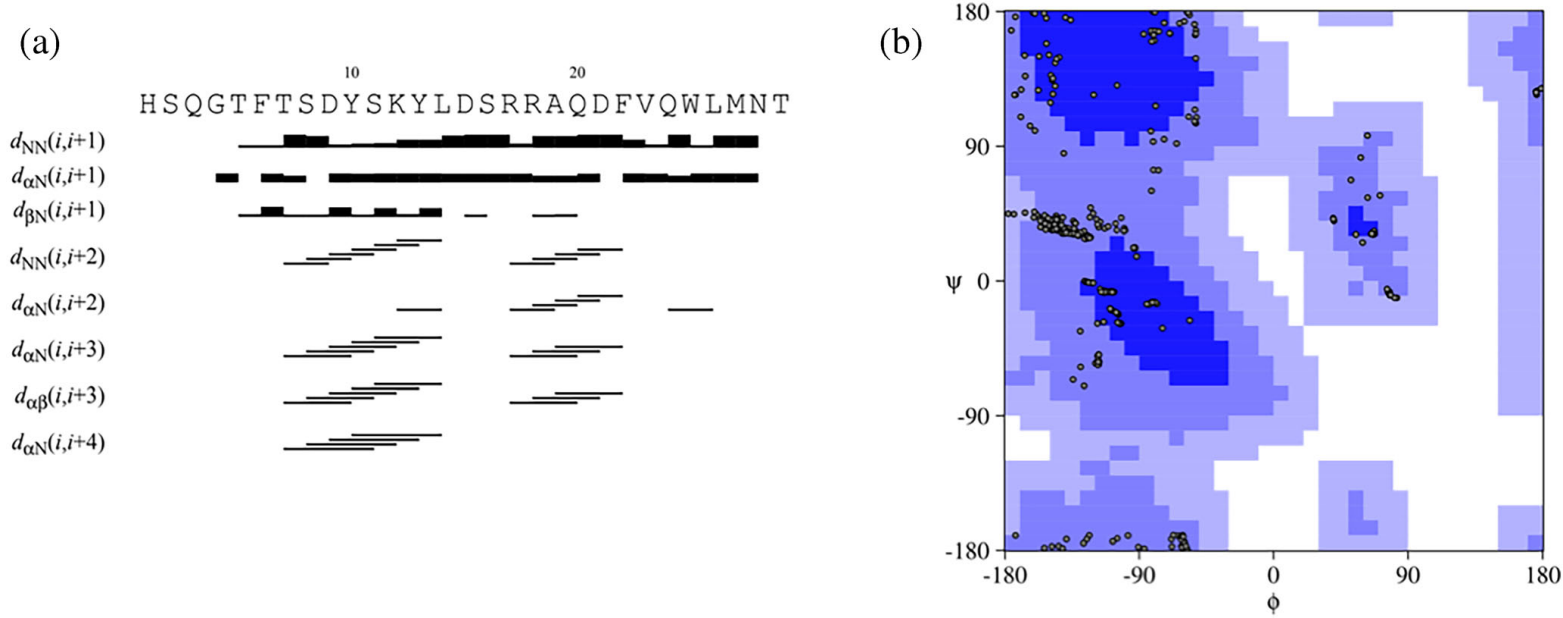
(a) Overview of the sequential and medium‐range NOEs used to calculate the overall structure of the glucagon peptide in H_2_O at pH 3.5 (Solution A); (b) Ramachandran plot showing the allowed and disallowed regions of the torsion angles of the glucagon peptide in water at pH 3.5 (Solution A).

Two‐dimensional NMR experiments (^1^H‐^1^H TOCSY and ^1^H‐^1^H NOESY) were performed on Solution A and on Formulation C. In Formulation B, the high lactose concentration masked glucagon resonances in the α‐proton region, preventing reliable structural assignment and calculation. To overcome this limitation, glucagon was reprepared at pH 2.5 without lactose, mimicking the formulation solvent system. This approach allowed us to verify whether conformational stabilization was due primarily to acidic conditions, as lactose is generally reported to act as a stabilizer of lyophilized powders rather than influencing peptide conformations in solution. Indeed, the analysis of CD, fluorescence, and superimposition of one‐dimensional spectra (Figures S3–S5) revealed no significant differences in spectral regions distant from lactose. For all acquired NMR spectra, proton resonance assignments were performed using the Wüthrich procedure (Wüthrich, 1986). According to this procedure, the identification and subsequent assignment of resonances associated with the spin systems of individual amino acids were performed by analyzing ^1^H‐^1^H‐TOCSY and ^1^H‐^1^H‐NOESY spectra. In Solution A, sequential and medium‐range NOEs (Figure 3a) indicated the presence of a short α‐helix spanning residues ^7^Thr‐Leu^14^, followed by a short loop and a 3_10_ helix from ^17^Arg‐Phe^22^. Ramachandran plot (Figure 3b) showed a broad φ/ψ distribution, indicating limited conformational constraints (Smith et al., 1996).

**FIGURE 4:**
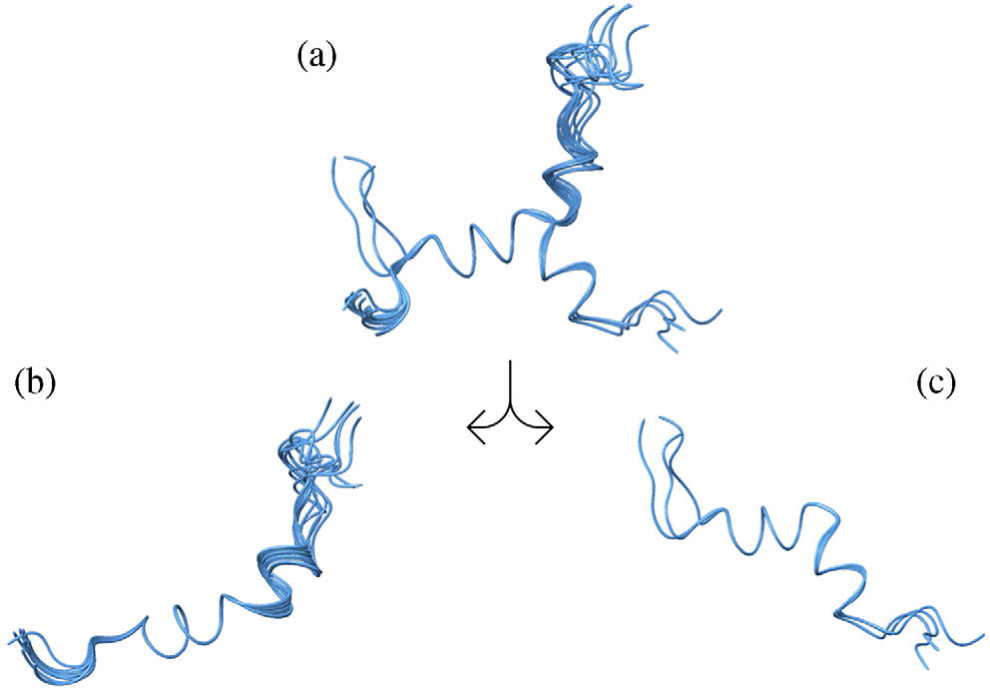
Bundle of the NMR structures of glucagon (Solution A) derived from data acquired in H_2_O at pH 3.5, at time t0 (a); structures in (b) and (c) represent the two main conformers.

For structural refinement, NOE‐derived peak volumes were converted into interproton distances. Ensembles were generated using simulated annealing molecular dynamics, and the most energetically favorable conformers were selected (Pearlman et al., 1995). The reproducibility of the structural calculations is judged based on the root mean square deviation (RMSD) values of atomic positions of each atom (Gottstein et al., 2012). The models included in the final bundle of structures calculated are reported in Figure 4a. The superposition on central residues (^11^Ser‐Arg^18^) indicates that the peptides within the bundle predominantly adopt two principal populations of flexible yet relatively ordered conformers (Figure 4b,c). This observation is attributable to the mobility of the loop comprising residues ^14^Leu‐Arg^18^, confirmed also by the absence of medium‐range connectivities in the bar plot in Figure 3a. Analysis of the H‐bonds in the bundle entries reveals that the interaction between Ser^8^ and Lys^12^ is highly prevalent. Additionally, other H‐bonds such as Ser^8^‐Asp^9^ and Asp^15^‐Ser^16^ are observed in the central region to a lesser extent (Figure S6A). The assignments of the proton resonances are reported in Table S1. Statistics for the final ensemble are summarized in Table S2. The structure was deposited in the Protein Data Bank (http://www.wwpdb.org/) with the PDB ID: 28TG.

The 3D structures were also calculated at t1–3 in this condition to have insights into the loss of stability from a structural point of view. After 1 day (t1, Figure S7A), the helix structure between ^7^Thr‐Leu^14^ was partially lost, and fewer sequential contacts were observed in the C‐terminal region (^24^Gln‐Thr^29^). Structures calculated for later time points (t2–t3) confirmed progressive disorder, characterized by the gradual loss of both short‐ and medium‐range connectivities (Figure S7B,C).

**FIGURE 5:**
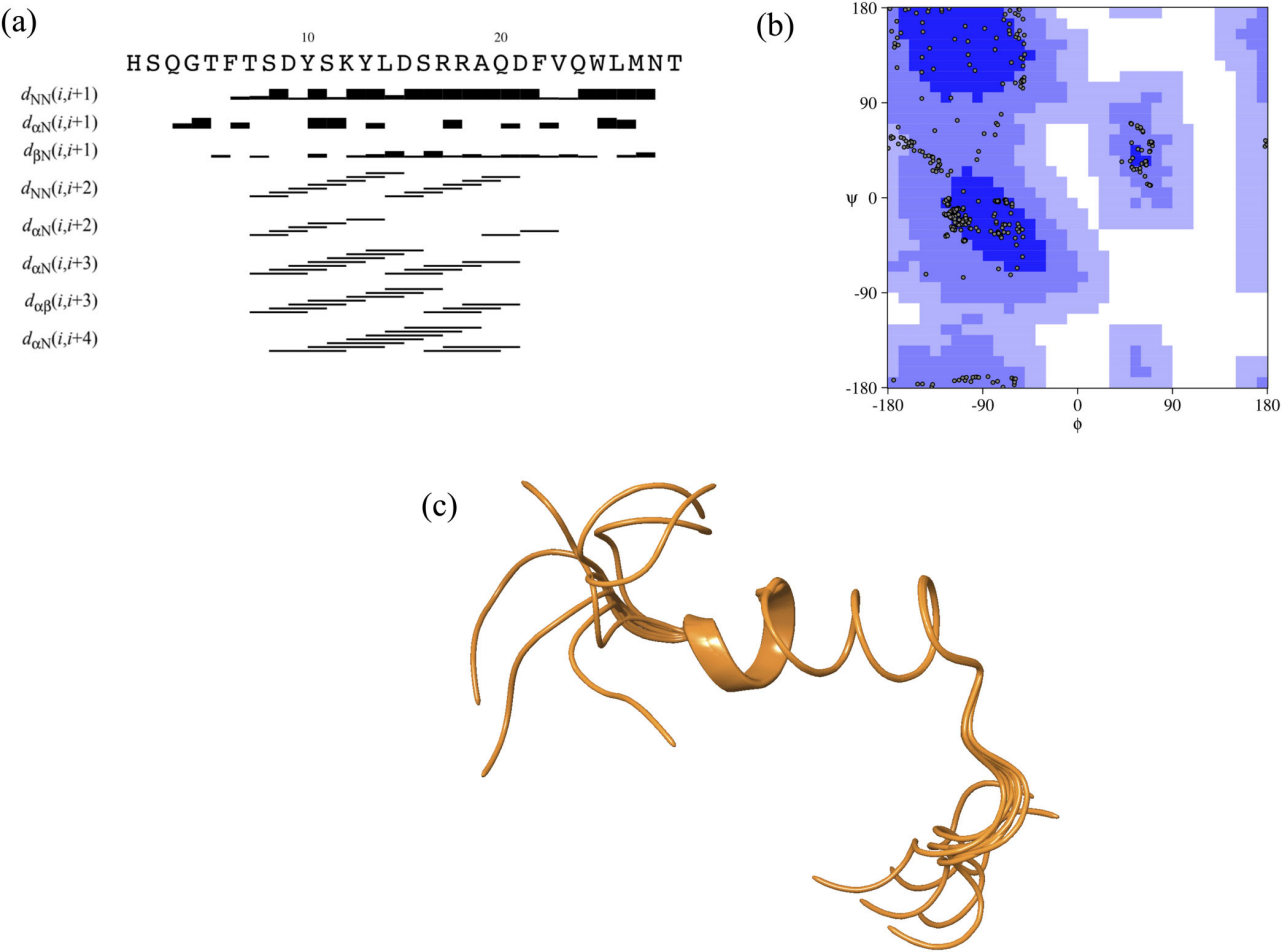
(a) Overview of the sequential and mid‐range NOEs used to calculate the ensemble structure of the glucagon peptide at pH 2.5; (b) Ramachandran plot providing an overview of the allowed and disallowed regions of the torsion angle values; (c) Bundle of the top 20 NMR structures of glucagon (Formulation B) based on the data acquired at pH 2.5.

Regarding glucagon at pH 2.5 (mimicking Formulation B), NOE connectivities (Figure 5a) revealed an α‐helix in the ^7^Thr‐Leu^14^ region, followed by a 3_10_ helix between ^15^Asp‐Asp^21^. The Ramachandran plot (Figure 5b) displayed a broad φ/ψ distribution, suggesting conformational flexibility. From 323 assigned NOE peaks, interproton distance restraints were obtained and used for structure calculations. The resulting ensemble is shown in Figure 5c. Analysis of the H‐bonds in the bundle entries reveals an overall higher number of interactions, with specifically frequent interactions involving the residues Thr^7^ and Ser^8^, which stabilize the α‐helix. In detail, H‐bonds between Thr^7^‐Tyr^10^, Thr^7^‐Ser^11^, Ser^8^‐Ser^11^, Ser^8^‐Lys^12^, Ser^8^‐Tyr^13^, Tyr^13^‐Arg^17^, Leu^14^‐Arg^18^, and Arg^17^‐Val^23^ are frequently observed (Figure S6B). The assignments of the proton resonances are reported in Table S3. Statistics for the final ensemble are summarized in Table S4. The structure was deposited in the Protein Data Bank (http://www.wwpdb.org/) with the PDB ID: 29CK.

**FIGURE 6:**
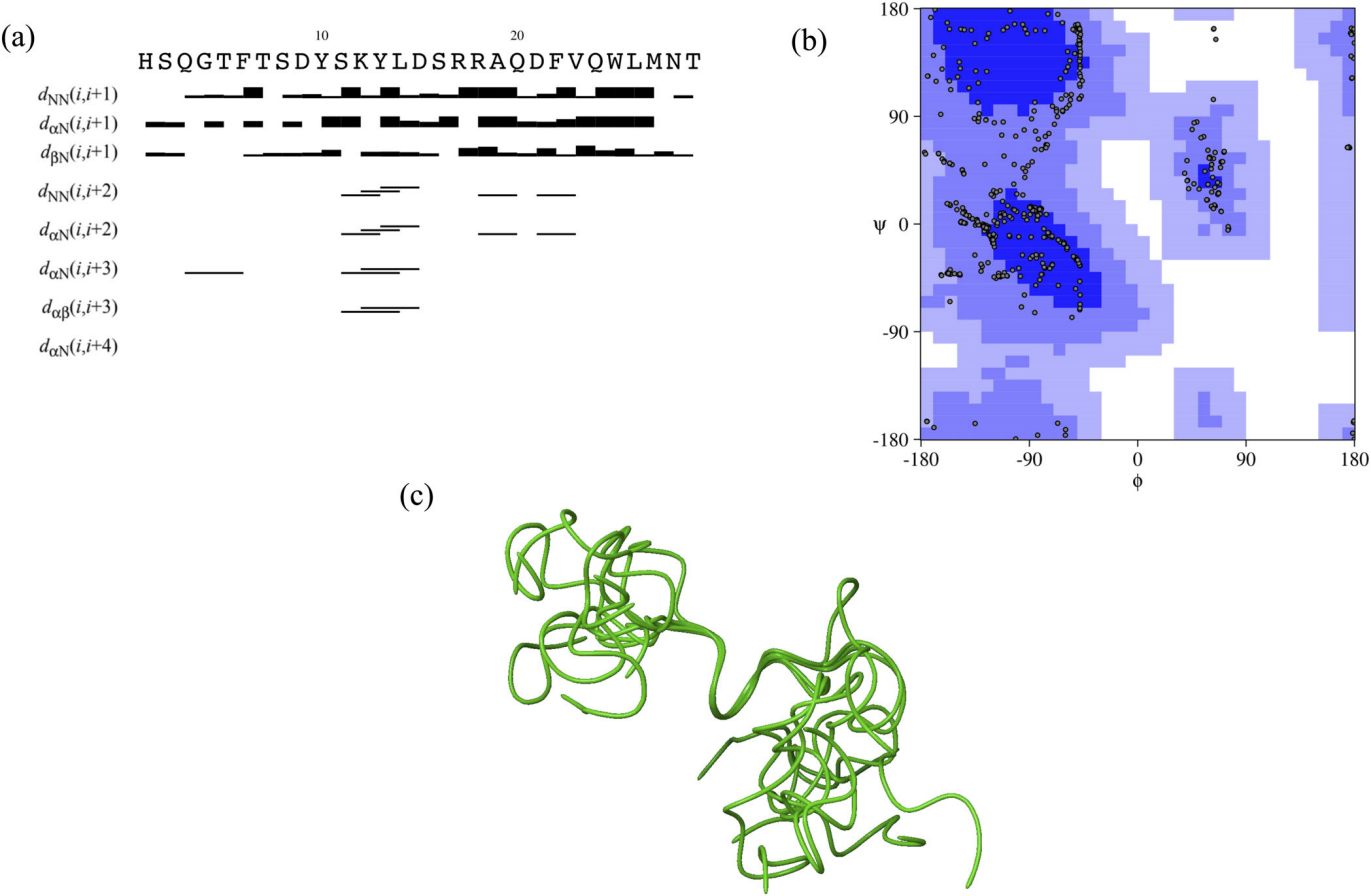
(a) Overview of the sequential and mid‐range NOEs used to calculate the ensemble structure of the glucagon peptide in DMSO_d6_ (Formulation C); (b) Ramachandran plot providing an overview of the allowed and disallowed regions of the torsion angle values; (c) Bundle of the NMR structures of glucagon (Formulation C) based on the data acquired in DMSO_d6_.

In Formulation C, correlations NN (i,i + 2), αN (i,i + 2), αN (i,i + 3), and αβ (i,i + 3) were observed (Figure 6a). These patterns indicated the presence of a short 3_10_ helix spanning residues ^11^Ser‐Asp^15^, followed by a type II turn between ^17^Arg‐Val^23^. The corresponding Ramachandran plot (Figure 6b) showed a broad φ/ψ distribution, consistent with the absence of rigid constraints that enforce a well‐defined secondary structure. For structural refinement, 264 NOE‐derived peaks were quantified, and their volumes were converted into interproton distance restraints. Structural calculations were carried out as described above, and the resulting ensemble is reported in Figure 6c. Analysis of the H‐bonds in the bundle entries reveals a lower number of interactions, with slightly more frequent interactions retrieved between residues Ser^8^‐Tyr^10^, Leu^14^‐Ser^16^, Ser^16^‐Gln^20^ and Gln^20^‐Val^23^ (Figure S6C). The assignments of the proton resonances are reported in Table S5. The statistics for the final NMR ensembles are reported in Table S6. The structure was deposited in the Protein Data Bank (http://www.wwpdb.org/) with the PDB ID: 28UX.

### 2.4 HPLC analysis

To complement the structural characterization, the chemical stability of glucagon in the three solvent systems was assessed by HPLC at the same three time points used for other analyses. Solution A showed a slight but progressive decrease in purity, with values of 98.33%, 97.68%, and 96.57%, respectively. Formulation B displayed a similar trend but with overall lower purity values: 96.71%, 94.89%, and 93.67%, suggesting a modest increase in degradation or chemical transformation over time. In contrast, Formulation C remained essentially unchanged throughout the experiment, with measured purities of 98.34%, 98.36%, and 98.37%, indicating high chemical stability over the monitored period (data not shown).

## 3 DISCUSSION

Stabilization of glucagon in solution is a major challenge due to its intrinsic tendency to undergo conformational transitions that can lead to aggregation. From a medicinal chemistry perspective, defining the structural elements that govern this behavior is essential for establishing structure–stability relationships that can inform rational formulation strategies. Structural characterization, therefore, provides a direct link between molecular architecture and macroscopic stability (Akbarian & Chen, 2022; Grimaldi et al., 2022).

In this study, glucagon was analyzed under three solvent systems with distinct physicochemical properties: an aqueous solution at pH 3.5 (Solution A), representing a metastable and aggregation‐prone environment; a clinically used lactose‐containing formulation at pH 2.5 (Formulation B); and a formulation composed of DMSO, trehalose, and sulfuric acid (Formulation C).

CD spectroscopy indicated that glucagon in Solution A initially adopts an α‐helical conformation that progressively evolves toward β‐sheet rich structures, consistent with an early aggregation pathway. In contrast, Formulation B maintained the α‐helical signature for several days, demonstrating that reduced pH, combined with carbohydrate excipients, effectively shifts the conformational equilibrium toward a soluble state. Fluorescence spectroscopy further supported these findings by showing a decrease in the intrinsic fluorescence of the peptide in Solution A. In contrast, the fluorescence signal remained stable in both formulations B and C, indicating reduced perturbation of the C‐terminal aromatic environment. To detect fibril formation over time, we performed a ThT assay. Solution A showed a rapid rise in ThT within 2–6 h, whereas formulations B and C remained flat up to 72 h, confirming the absence of early‐stage aggregates. Under accelerated thermomechanical stress (37°C, 900 rpm), Solution A collapsed within 30 min, and tracking the tryptophan blue shift revealed a clear divergence under such conditions. Formulation B gradually exhibited a blue shift over 48 h, whereas Formulation C demonstrated high stability, indicating that such formulation effectively preserves the peptide structural conformation over time. Residue‐specific information obtained from solution NMR revealed that conformational destabilization in Solution A primarily involves the central helical region (^7^Thr‐Leu^14^) and the C‐terminal segment (^24^Gln‐Thr^29^).

The analyses at pH 3.5 were performed near the pKa of the aspartate residues, which can cause conformational instability. Bundle analysis at this pH identified two distinct conformations. One showed a low RMSD of 0.0628 Å for residues ^7^Thr‐Leu^14^ and 0.0169 Å for residues ^18^Arg‐Asp^21^, indicating two helix structures within the ensemble linked by a flexible loop from ^15^Asp‐Arg^17^. After 1 day, RMSD for residues ^7^Thr‐Leu^14^ increased to 2.33 Å, while it remained very low (0.00680 Å) for residues ^18^Arg‐Asp^21^. This suggests that the first helix segment destabilizes significantly, whereas the ^18^Arg‐Asp^21^ region remains more stable, aligning with trends observed in the sequence plots. These regions, therefore, represent key structural determinants in the early stages of aggregation. In contrast, at pH 2.5, which replicates Formulation B, a sustained network of intramolecular interactions was preserved, particularly across the ^7^Thr‐Val^23^ segment, suggesting that protonation states and excipient interactions modulate the stability of this region. In Formulation C, glucagon assumed a flexible but defined conformation characterized by a short helix segment in the central moiety (^11^Ser‐Leu^14^), highlighting the contribution of solvent polarity and reduced water activity to suppress conformational fluctuations.

Collectively, these data establish two primary stability‐determining regions: a central motif encompassing residues ^11^Ser‐Leu^14^ that remains conformationally constrained across all conditions, and a C‐terminal domain that exhibits high sensitivity to solvent‐dependent destabilization. This structure–stability relationship is consistent with solid‐state NMR studies of glucagon fibrils, which show that the central and C‐terminal regions adopt β‐strand‐rich conformations and function as aggregation‐prone motifs within the cross‐β framework (Gelenter et al., 2019). At the residue level, polar side chains located in central regions (Thr^7^, and Ser^11^) appear to favor intramolecular hydrogen bonding and stabilization of regular secondary structure, thereby opposing conversion to β‐sheet geometries. In contrast, fibril stabilization is driven by a complementary network of hydrophobic and polar interactions. Aromatic and aliphatic residues (Phe^6^, Tyr^10^, Tyr^13^, Phe^22^, Trp^25^, Leu^26^, and Met^27^) form a hydrophobic core that promotes interstrand packing, while charged and polar residues (Gln^3^, Asp^9^, Asp^15^, Arg^17^, Arg^18^, Asp^21^, and Gln^24^) establish intermolecular hydrogen bonds that stabilize β‐sheet alignment. These cooperative interactions define the molecular interaction pattern underlying the cross‐β architecture of glucagon fibrils (Gelenter et al., 2019).

These findings support a dual conformational profile for glucagon that depends on its chemical environment. In membrane‐like or micellar systems, glucagon preferentially adopts α‐helical conformations, consistent with its receptor‐bound structure in lipid membranes (PDB ID: 5YQZ) (Zhang et al., 2018). In aqueous environments, by contrast, glucagon adopts conformations that favor β‐strand formation and subsequent amyloid assembly (Braun et al., 1983; Ying et al., 2003). This equilibrium between helical and β‐prone states can therefore be modulated by solvent composition, pH, and excipient chemistry, providing leverage points for formulation design.

HPLC analysis demonstrated that all three systems maintained high chemical integrity for 48 h, with only minor purity losses observed in aqueous samples. Notably, these variations in chemical stability did not parallel the pronounced conformational differences detected by CD and NMR. In Solution A, extensive loss of secondary structure occurred despite minimal chemical degradation, indicating that aggregation is driven primarily by conformational reorganization rather than by covalent peptide modification. Conversely, Formulation B displayed slightly reduced chemical stability while maintaining a stable conformational profile, further illustrating that chemical stability and conformational stability are not necessarily correlated. Formulation C exhibited excellent chemical stability in DMSO and strongly suppressed conformational transitions, consistent with reduced peptide–peptide association in this solvent environment.

Overall, this study defines a structure–stability relationship for glucagon in which aggregation propensity is controlled by solvent‐dependent modulation of specific structural motifs, particularly within the central and C‐terminal regions. By identifying the molecular features that stabilize helix conformations and suppress β‐sheet formation, these results provide a mechanistic basis for the rational selection of solvent systems and excipients that favor soluble glucagon states. From a medicinal chemistry standpoint, this knowledge enables the design of formulations and delivery platforms that maintain glucagon in its therapeutically active, non‐aggregated form and supports the development of improved preparations for advanced applications such as dual‐hormone delivery systems.

## 4 MATERIALS AND METHODS

### 4.1 Sample preparation

Glucagon samples (lyophilized powder kindly provided by Fresenius Kabi Italia S.r.l.) were prepared under three different conditions: (i) Solution A: 1 mg/mL glucagon dissolved in water (aqueous solution at physiological pH); (ii) Formulation B: 1 mg/mL glucagon dissolved in water containing 0.01 M HCl and 107 mg/mL lactose. For NMR structural studies, lactose was omitted to avoid signal masking; (iii) Formulation C: 5 mg/mL glucagon dissolved in dimethyl sulfoxide (DMSO_d6_) containing 55 mg/mL trehalose dihydrate and 6 mg/mL 1 N H_2_SO₄. Aqueous samples (A and B) were filtered through a 0.45 μm filter prior to use to prevent the formation of pre‐formed aggregates. For NMR experiments, 10% D_2_O was added to aqueous samples.

### 4.2 Circular dichroism (CD) spectra acquisition

CD spectra were obtained using a JASCO J‐810 spectropolarimeter, with the aid of a 1 cm long quarter cell and working at a temperature of 25°C. CD spectra were obtained by averaging 4 scans, in a measurement range 260–190 nm, at a bandwidth of 1 nm and a scanning speed of 10 nm/min (Santoro et al., 2023). Each spectrum was processed by subtracting the solvent spectrum. For the estimation of secondary structure contents, CD spectra were analyzed using the CONTIN algorithm of the DICHROWEB program available online (Ciaglia et al., 2024; Whitmore & Wallace, 2008). All CD spectra were acquired with a peptide concentration of 50 μM.

### 4.3 Acquisition of fluorescence spectra

Intrinsic fluorescence was measured at 25°C on a Perkin Elmer LS55 spectrofluorimeter. Excitation was set at 280 nm, and emission spectra were collected from 260 to 450 nm using a 5 nm excitation slit, 2.5 nm emission slit, and a scan speed of 200 nm/min. Data were analyzed using the Spectragryph software (Menges, n.d.). Detection of early‐stage aggregation nuclei was performed using the fluorophore ThT. Aliquots of Solution A, Formulation B, and Formulation C were sampled at predetermined intervals: t0, 2 h, 6 h, 24 h, 48 h, and 72 h. Fluorescence emission spectra were recorded at 25°C on a spectrofluorimeter, with excitation at 450 nm and emission from 460 to 585 nm (Santoro et al., 2022). Raw data were baseline‐corrected and smoothed for graphical presentation. To evaluate the long‐term robustness of the formulations, samples were sealed in vials, incubated at 37°C, and subjected to continuous mechanical agitation at 900 rpm for 48 h (Stimple et al., 2019). The structural status of glucagon was monitored by intrinsic fluorescence spectroscopy at specific time points (t0, 30 min, 1 h, 2 h, 4 h, 6 h, 12 h, 24 h, and 48 h).

### 4.4 Acquisition of NMR spectra

#### 4.4.1 One‐ and two‐dimensional experiments

NMR spectra were recorded using the Bruker DRX600 spectrometer, a 600 MHz magnet equipped with a 5 mm triple resonance probe (TXI) and z‐gradient. All experiments were acquired at 25°C. One‐dimensional ^1^H and two‐dimensional homonuclear spectra were acquired using sequences that suppress the water signal via excitation sculpting (Parella et al., 1998). The 2D homonuclear ^1^H‐^1^H‐TOCSY and ^1^H‐^1^H‐NOESY experiments were acquired in phase sensitive mode using quadrature detection in ω1 by phase stepping proportional to the initial pulse time (Bax & Davis, 1985; Jeneer et al., 1979). For the ^1^H‐^1^H‐TOCSY experiments, a mixing time of 80 ms was used; for the ^1^H‐^1^H‐NOESY experiments, a mixing time of 100 ms. All spectra were processed by Fourier transformation, with quadrature detection.

#### 4.4.2 Structure calculation

The integrated volumes of the ^1^H‐^1^H NOESY cross‐peaks were converted into XEASY peak format to enable the derivation of intramolecular distance restraints for the calculation of the three‐dimensional structure of glucagon under various conditions using CYANA 2.1. Distance restraints were computed by translating peak volumes into upper‐bound limits using the CALIBA macro (Güntert, 2004; Orlandin et al., 2025). Redundant and duplicate restraints were systematically removed to yield a refined list of non‐redundant constraints (Buonocore et al., 2022). This curated dataset was employed to generate an ensemble of 20 conformers via a torsion‐angle simulated annealing protocol comprising 50,000 steps. Structural models were ranked based on the lowest target function values (ranging from 2 to 20 Å), and the most representative structures were subsequently visualized and analyzed using Schrödinger's Maestro software (version 12.5.139.33) (Maestro, 2020).

## AUTHOR CONTRIBUTIONS

**Marco Macis:** Validation; formal analysis; investigation. **Antonio Ricci:** Conceptualization; resources; writing – review and editing; project administration. **Angelo Santoro:** Conceptualization; methodology; validation; formal analysis; investigation; data curation; writing – original draft; writing – review and editing; visualization. **Anna Maria D'Ursi:** Conceptualization; methodology; resources; writing – review and editing; supervision; project administration. **Michela Buonocore:** Validation; formal analysis; investigation; data curation; writing – original draft; writing – review and editing; visualization; project administration.

## CONFLICT OF INTEREST STATEMENT

The authors declare no conflicts of interest.

## Supporting information


**Figure S1.** ThT fluorescence profiles under static conditions over 72 h of (A) Solution A (blue gradient), (B) Formulation B (orange gradient), and (C) Formulation C (green gradient).
**Figure S2.** Intrinsic fluorescence spectra under accelerated thermo‐mechanical stress (37°C, 900 rpm) over 48 h of (A) Solution A (blue gradient), (B) Formulation B (orange gradient), and (C) Formulation C (green gradient).
**Figure S3.** CD curves and secondary structure quantification performed with the CONTIN algorithm of (A) glucagon in water and lactose at pH 2.5 (Formulation B) and (B) of glucagon in water at pH 2.5, at system establishment (t0) and after one (t1), two (t2) and three (t3) days.
**Figure S4.** Fluorescence curves of (A) glucagon in water and lactose at pH 2.5 (Formulation B) and (B) of glucagon in water at pH 2.5, at system establishment (t0) and after one (t1), two (t2) and three (t3) days.
**Figure S5.** Superimposition of glucagon at pH 2.5 (black) and at pH 2.5 in presence of lactose (red).
**Figure S6.** Heatmap showing the frequency of intramolecular H‐bonds between glucagon residues in the bundles of NMR structures obtained from (A) Solution A, (B) Formulation B, and (C) Formulation C.
**Table S1.** ^1^H Chemical Shifts of glucagon in H_2_O at pH 3.5 (Solution A) at t0.
**Table S2.** Statistics for the structural calculation of the NMR ensemble of glucagon in water, at pH 3.5 (Solution A) at t0.
**Figure S7.** Bundle of the NMR structures and overview of the sequential and medium‐range NOEs used to calculate the overall structure of glucagon based on NMR data acquired in H_2_O at pH 3.5 (Solution A), at time t0 (A), after 1 day (t1, B), after 2 days (t2, C) and after 3 days (t3, D).
**Table S3.** ^1^H Chemical Shifts of glucagon in H_2_O at pH 2.5 (Formulation B).
**Table S4.** Statistics for the structural calculation of the NMR ensemble of glucagon in water, at pH 2.5 (mimicking Formulation B).
**Table S5.** ^1^H Chemical Shifts of glucagon in DMSO_d6_ (Formulation C).
**Table S6.** Statistics for the structural calculation of the NMR ensemble of glucagon in DMSO_d6_ (Formulation C).

## Data Availability

The data that support the findings of this study are available from the corresponding author upon reasonable request.
