# Supplementary material for: Conformational determinants of glucagon stability and aggregation kinetics in aqueous and commercial formulations
Source: Protein Sci. 2026 Aug 5;35(9):e70750. doi: 10.1002/pro.70750 (PMC13443137; doi:10.1002/pro.70750)
Supplement: Supplementary file 1 [file PRO-35-e70750-s001.docx]

**Conformational determinants of glucagon stability and aggregation kinetics in aqueous and commercial formulations**

**Supporting Materials**

Angelo Santoro^1^, Marco Macis^2^, Michela Buonocore^3,*^, Antonio Ricci^2,*^, Anna Maria D’Ursi^1^

^1^ Department of Pharmacy, University of Salerno, Via Giovanni Paolo II, 132, Fisciano, 84084 Salerno, Italy;
^2^ Fresenius Kabi iPSUM, Via Roma, 108, Cassina de' Pecchi, 20051 Milano, Italy;
^3^ Department of Chemical Sciences, University of Naples “Federico II”, Complesso Universitario Monte Sant’Angelo, Via Cinthia, 80126 Napoli, Italy
^*^ Authors to whom correspondence should be addressed

**Table of Contents**

**Figure S1.** ThT fluorescence profiles under static conditions over 72 h of A) Solution A (blue gradient), B) formulation B (orange gradient), and C) Formulation C (green gradient).

**Figure S2.** Intrinsic fluorescence spectra under accelerated thermo-mechanical stress (37°C, 900 rpm) over 48 h of A) Solution A (blue gradient), B) Formulation B (orange gradient), and C) Formulation C (green gradient).

**Figure S3.** CD curves and secondary structure quantification performed with the CONTIN algorithm of **A**) glucagon in water and lactose at pH 2.5 (formulation B) and **B**) of glucagon in water at pH 2.5, at system establishment (t0) and after one (t1), two (t2) and three (t3) days.

**Figure S4.** Fluorescence curves of **A**) glucagon in water and lactose at pH 2.5 (formulation B) and **B**) of glucagon in water at pH 2.5, at system establishment (t0) and after one (t1), two (t2) and three (t3) days.

**Figure S5.** Superimposition of glucagon at pH 2.5 (black) and at pH 2.5 in presence of lactose (red).

**Figure S6.** Heatmap showing the frequency of intramolecular H-bonds between glucagon residues in the bundles of NMR structures obtained from (**A**) solution A, (**B**) formulation B, and (**C**) formulation C.

**Table S1.** ^1^H Chemical Shifts of glucagon in H_2_O at pH 3.5 (solution A).

**Table S2.** Statistics for the structural calculation of the NMR ensemble of glucagon in water, at pH 3 (solution A) at t0.

**Figure S7.** Bundle of the top 20 NMR structures and overview of the sequential and medium-range NOEs used to calculate the overall structure of glucagon (Solution A) based on NMR data acquired in H_2_O at pH 3.5, at time t0 (**A**), after one day t1 (**B**), after two days (**C**) and after three days (**D**).

**Table S3.** ^1^H Chemical Shifts of glucagon in H_2_O at pH 2.5 (formulation B).

**Table S4.** Statistics for the structural calculation of the NMR ensemble of glucagon in water, at pH 2.5 (mimicking of formulation B).

**Table S5.** ^1^H Chemical Shifts of glucagon in DMSO_d6_ (formulation C).

**Table S6.** Statistics for the structural calculation of the NMR ensemble of glucagon in DMSO_d6_ (formulation C).

**Figure S1.** ThT fluorescence profiles under static conditions over 72 h of A) Solution A (blue gradient), B) formulation B (orange gradient), and C) Formulation C (green gradient).

**Figure S2.** Intrinsic fluorescence spectra under accelerated thermo-mechanical stress (37°C, 900 rpm) over 48 h of A) Solution A (blue gradient), B) Formulation B (orange gradient), and C) Formulation C (green gradient).

**Figure S3.** CD curves and secondary structure quantification performed with the CONTIN algorithm of **A**) glucagon in water and lactose at pH 2.5 (formulation B) and **B**) of glucagon in water at pH 2.5, at system establishment (t0) and after one (t1), two (t2) and three (t3) days.

**Figure S4.** Fluorescence curves of **A**) glucagon in water and lactose at pH 2.5 (formulation B) and **B**) of glucagon in water at pH 2.5, at system establishment (t0) and after one (t1), two (t2) and three (t3) days.

**Figure S5.** Superimposition of glucagon at pH 2.5 (black) and at pH 2.5 in presence of lactose (red).

**Figure S6.** Heatmap showing the frequency of intramolecular H-bonds between glucagon residues in the bundles of NMR structures obtained from (**A**) solution A, (**B**) formulation B, and (**C**) formulation C.

**Table S1.** ^1^H Chemical Shifts of glucagon in H_2_O at pH 3.5 (solution A) at t0.

| **Residue** | **HN** | **HA** | **HB** | **HG** | **HD** | **HE** |
| --- | --- | --- | --- | --- | --- | --- |
| ^1^His | / | / | / |  | / |  |
| ^2^Ser | 8.78 | 4.46 | 3.79 |  |  |  |
| ^3^Gln | 8.68 | 4.34 | 2.06  1.95 | 2.32 |  |  |
| ^4^Gly | 8.39 | 3.89 |  |  |  |  |
| ^5^Thr | 7.92 | 4.24 | 4.03 | 1.01 |  |  |
| ^6^Phe | 8.27 | 4.60 | 3.03  2.91 |  | / |  |
| ^7^Thr | 8.00 | 4.20 | 4.13 | 1.08 |  |  |
| ^8^Ser | 8.20 | 4.30 | 3.79  3.73 |  |  |  |
| ^9^Asp | 8.27 | 4.51 | 2.64  2.58 |  |  |  |
| ^10^Tyr | 7.95 | 4.33 | 2.98  2.93 |  |  |  |
| ^11^Ser | 8.04 | 4.09 | 3.79  3.73 |  |  |  |
| ^12^Lys | 7.81 | 4.04 | 1.60 | 1.14 | 1.52 | 2.82 |
| ^13^Tyr | 7.76 | 4.29 | 2.97  2.76 |  |  |  |
| ^14^Leu | 7.84 | 3.99 | 1.40 | 1.38 | 0.74  0.70 |  |
| ^15^Asp | 8.00 | 4.49 | 2.68 |  |  |  |
| ^16^Ser | 8.05 | 4.25 | 3.87 |  |  |  |
| ^17^Arg | 8.05 | 4.16 | 1.75  1.67 | 1.53  1.49 | 3.02 |  |
| ^18^Arg | 8.01 | 4.14 | 1.75  1.67 | 1.52  1.49 | 3.00 |  |
| ^19^Ala | 8.12 | 4.12 | 1.32 |  |  |  |
| ^20^Gln | 8.15 | 4.13 | 1.97 | 2.26 |  |  |
| ^21^Asp | 8.09 | 4.50 | 2.63 |  |  |  |
| ^22^Phe | 7.95 | 4.34 | 2.97 |  |  |  |
| ^23^Val | 7.79 | 3.72 | 1.85 | 0.68 |  |  |
| ^24^Gln | 8.01 | 4.02 | 1.89 | 2.18 |  |  |
| ^25^Trp | 8.11 | 4.33 | 2.88 |  |  |  |
| ^26^Leu | 7.89 | 4.21 | 1.40 | 0.70  0.68 |  |  |
| ^27^Met | 7.88 | 4.25 | 1.97  1.89 | 2.48 |  |  |
| ^28^Asn | 7.88 | 4.45 | 3.22  3.13 |  |  |  |
| ^29^Thr | 7.61 | 4.11 | 4.08 | 0.98 |  |  |

**Table S2.** Statistics for the structural calculation of the NMR ensemble of glucagon in water, at pH 3.5 (solution A) at t0.

| *Number of experimental restraints after CYANA* | |
| --- | --- |
| Total NOEs | 299 |
| Intra-residual | 207 |
| Sequential | 56 |
| Long-range | 36 |
| *Ramachandran Analysis* | |
| Favorable regions | *74.6%* |
| Additional allowed regions | *25.4%* |
| Generously allowed regions | *0%* |
| Disallowed regions | *0%* |

**Figure S7.** Bundle of the NMR structures and overview of the sequential and medium-range NOEs used to calculate the overall structure of glucagon based on NMR data acquired in H_2_O at pH 3.5 (Solution A), at time t0 (**A**), after one day (t1, **B**), after two days (t2, **C**) and after three days (t3, **D**).

**Table S3.** ^1^H Chemical Shifts of glucagon in H_2_O at pH 2.5 (formulation B).

| **Residue** | **HN** | **HA** | **HB** | **HG** | **HD** | **HE** |
| --- | --- | --- | --- | --- | --- | --- |
| ^1^His | / | / | / |  | / |  |
| ^2^Ser | 8.78 | 4.46 | 3.80 |  |  |  |
| ^3^Gln | 8.67 | 4.32 | 2.07  1.96 | 2.31 |  |  |
| ^4^Gly | 8.38 | 3.90 |  |  |  |  |
| ^5^Thr | 7.95 | 4.23 | 4.23 | 1.03 |  |  |
| ^6^Phe | 8.29 | 4.62 | 3.04  2.93 |  | / |  |
| ^7^Thr | 8.00 | 4.22 | 4.11 | 1.09 |  |  |
| ^8^Ser | 8.22 | 4.28 | 3.80  3.75 |  |  |  |
| ^9^Asp | 8.17 | 4.71 | 2.66  2.63 |  |  |  |
| ^10^Tyr | 7.99 | 4.30 | 2.99  2.93 |  |  |  |
| ^11^Ser | 8.01 | 4.14 | 3.78  3.72 |  |  |  |
| ^12^Lys | 7.87 | 4.02 | 1.61 | 1.15 | 1.53 | 2.83 |
| ^13^Tyr | 7.80 | 4.26 | 2.95  2.76 |  |  |  |
| ^14^Leu | 7.85 | 3.99 | 1.44 | 1.40 | 0.75  0.71 |  |
| ^15^Asp | 8.12 | 4.55 | 2.86  2.75 |  |  |  |
| ^16^Ser | 8.00 | 4.30 | 3.78 |  |  |  |
| ^17^Arg | 8.04 | 4.16 | 1.74  1.65 | 1.53  1.48 | 3.03 |  |
| ^18^Arg | 8.02 | 4.16 | 1.74  1.65 | 1.52  1.48 | 2.99 |  |
| ^19^Ala | 8.13 | 4.15 | 1.31 |  |  |  |
| ^20^Gln | 8.14 | 4.18 | 1.90 | 2.23 |  |  |
| ^21^Asp | 8.23 | 4.58 | 2.76 |  |  |  |
| ^22^Phe | 8.07 | 4.36 | 2.91 |  |  |  |
| ^23^Val | 7.80 | 3.78 | 1.81 | 0.67 |  |  |
| ^24^Gln | 8.05 | 4.06 | 1.85 | 2.14 |  |  |
| ^25^Trp | 8.00 | 4.39 | 2.93 |  |  |  |
| ^26^Leu | 7.91 | 4.26 | 1.37 | 0.72  0.69 |  |  |
| ^27^Met | 7.91 | 4.21 | 1.95  1.88 | 2.48 |  |  |
| ^28^Asn | 7.92 | 4.51 | 3.23  3.14 |  |  |  |
| ^29^Thr | 7.57 | 4.11 | 4.07 | 1.02 |  |  |

**Table S4.** Statistics for the structural calculation of the NMR ensemble of glucagon in water, at pH 2.5 (mimicking formulation B).

| *Number of experimental restraints after CYANA* | |
| --- | --- |
| Total NOEs | 323 |
| Intra-residual | 222 |
| Sequential | 51 |
| Long-range | 50 |
| *Ramachandran Analysis* | |
| Favorable regions | 58.7% |
| Additional allowed regions | 37.7% |
| Generously allowed regions | 3.7% |
| Disallowed regions | 0.0% |

**Table S5.** ^1^H Chemical Shifts of glucagon in DMSO_d6_ (formulation C).

| **Residue** | **HN** | **HA** | **HB** | **HG** | **HD** | **HE** |
| --- | --- | --- | --- | --- | --- | --- |
| ^1^His | 8.28 | 4.18 | 3.22 |  | 6.97 | 8.78 |
| ^2^Ser | 8.71 | 4.39 | 3.69  3.64 |  |  |  |
| ^3^Gln | 8.51 | 4.31 | 1.94  1.80 | 2.16 |  |  |
| ^4^Gly | 8.16 | 3.82  3.73 |  |  |  |  |
| ^5^Thr | 7.77 | 4.22 | 3.90 | 0.97 |  |  |
| ^6^Phe | 8.06 | 4.62 | 3.07  2.83 |  | 7.15 | 7.22 |
| ^7^Thr | 8.00 | 4.28 | 3.97 | 1.04 |  |  |
| ^8^Ser | 7.90 | 4.39 | 3.68  3.59 |  |  |  |
| ^9^Asp | 8.34 | 4.51 | 2.60  2.44 |  |  |  |
| ^10^Tyr | 7.80 | 4.38 | 2.87  2.65 |  | 6.95 | 6.66 |
| ^11^Ser | 7.99 | 4.28 | 3.63  3.57 |  |  |  |
| ^12^Lys | 8.02 | 4.21 | 1.49 | 1.48 | 1.22 | 2.72 |
| ^13^Tyr | 7.82 | 4.37 | 2.91  2.63 |  | 6.95 | 6.66 |
| ^14^Leu | 7.94 | 4.27 | 1.46 | 1.56 | 0.86  0.81 |  |
| ^15^Asp | 8.16 | 4.57 | 2.71  2.51 |  |  |  |
| ^16^Ser | 7.71 | 4.28 | 3.61  3.52 |  |  |  |
| ^17^Arg | 8.06 | 4.24 | 1.71 | 1.54 | 3.08 |  |
| ^18^Arg | 8.01 | 4.24 | 1.70 | 1.50 | 3.08 |  |
| ^19^Ala | 8.01 | 4.18 | 1.21 |  |  |  |
| ^20^Gln | 7.93 | 4.18 | 1.84  1.72 | 2.08 |  |  |
| ^21^Asp | 8.12 | 4.51 | 2.62  2.43 |  |  |  |
| ^22^Phe | 7.85 | 4.55 | 3.00  2.81 |  | 7.14 | 7.22 |
| ^23^Val | 7.92 | 4.07 | 1.92 | 0.77 |  |  |
| ^24^Gln | 8.03 | 4.22 | 1.84  1.72 | 2.10 |  |  |
| ^25^Trp | 8.02 | 4.54 | 3.10  2.94 |  | 7.16 | 7.41 |
| ^26^Leu | 8.03 | 4.26 | 1.44 | 1.58 | 0.84  0.81 |  |
| ^27^Met | 8.00 | 4.33 | 1.92  1.80 | 2.46  2.44 |  | 1.99 |
| ^28^Asn | 8.18 | 4.64 | 2.57  2.42 |  |  |  |
| ^29^Thr | 7.55 | 4.16 | 4.12 | 1.03 |  |  |

**Table S6.** Statistics for the structural calculation of the NMR ensemble of glucagon in DMSO_d6_ (formulation C).

| *Number of experimental restraints after CYANA* | |
| --- | --- |
| Total NOEs | 263 |
| Intra-residual | 181 |
| Sequential | 67 |
| Long-range | 15 |
| *Ramachandran Analysis* | |
| Favorable regions | 37.5% |
| Additional allowed regions | 53.3% |
| Generously allowed regions | 8.7% |
| Disallowed regions | 0.6% |
